# Dissecting and Evaluating the Therapeutic Targets of Coptis Chinensis Franch in the Treatment of Urinary Tract Infections Induced by *Escherichia coli*

**DOI:** 10.3389/fphar.2021.794869

**Published:** 2022-01-12

**Authors:** Zhenglin Chang, Jinhu Zhang, Min Lei, Zheng Jiang, Xiangkun Wu, Yapeng Huang, Zhican He, Yuyan Zhang, Shujue Li, Xiaolu Duan, Wenqi Wu

**Affiliations:** ^1^ Guangdong Key Laboratory of Urology, Department of Urology, The First Affiliated Hospital of Guangzhou Medical University, Guangzhou, China; ^2^ Department of Urology, The Second Affiliated Hospital, Guangzhou Medical University, Guangzhou, China; ^3^ Department of Pathology, Nanfang Hospital and Basic Medical College, Southern Medical University, Guangzhou, China; ^4^ Guangzhou Institute of Dermatology, Guangzhou, China

**Keywords:** anti-inflammatory, molecular docking, network pharmacology, UTIs, coptis chinensis Franch

## Abstract

Coptis chinensis Franch (CCF) is extensively used in the treatment of inflammatory-related diseases. Accumulating studies have previously demonstrated the anti-inflammatory properties of CCF, yet data on its exact targets against urinary tract infections (UTIs) remain largely unknown. Therefore, the present study decodes the potential targets of action of CCF against UTIs by network pharmacology combined with experiment evaluations. Based on the pharmacology network analysis, the current study yielded six core ingredients: quercetin, palmatine (R)-canadine, berlambine, berberine, and berberrubine. The protein–protein interaction network (PPI) was generated by the string database, and then, four targets (IL6, FOS, MYC, and EGFR) were perceived as the major CCF targets using the CytoNCA plug-in. The results of molecular docking showed that the six core constituents of CCF had strong binding affinities toward the four key targets of UTIs after docking into the crystal structure. The enrichment analysis indicated that the possible regulatory mechanisms of CCF against UTIs were based on the modules of inflammation, immune responses, and apoptosis among others. Experimentally, the *Escherichia coli* (*E. coli*) strain CFT073 was applied to establish *in vivo* and *in vitro* models. *In vivo* results revealed that the key targets, IL6 and FOS, are significantly upregulated in rat bladder tissues of UTIs, whereas the expression of MYC and EGFR remained steady. Last, *in vitro* results further confirmed the therapeutic potential of CCF by reducing the expression of IL6 and FOS. In conclusion, IL6 and FOS were generally upregulated in the progression of *E. coli–induced* UTIs, whereas the CCF intervention exerted a preventive role in host cells stimulated by *E. coli*, partially due to inhibiting the expression of IL6 and FOS.

## Introduction

Urinary tract infections (UTIs) are regarded to be among the most common bacterial infections, affecting 150 million people around the world annually (Sihra et al., 2018; Klein and Hultgren, 2020; Behzadi et al., 2021b). Data estimates show that up to 60% of females report a history of at least one UTI, and more than 50% of the women are affected by a recurrent infection after 6 months (Fisher et al., 2018; Sihra et al., 2018). As the leading cause of UTIs, *Escherichia coli* (*E. coli*) infections typically occur in the bladder (cystitis) and may ascend to the kidney, resulting in pyelonephritis (Hozzari et al., 2020; Klein and Hultgren, 2020). In some severe cases, *E. coli–related* UTIs can progress to chronic renal failure or urosepsis, which may be fatal (Chamoun et al., 2020; Klein and Hultgren, 2020). Virulence factors are the arsenal of bacteria, such as type P, type 1, type 3 fimbriae, etc. (Khonsari et al., 2021). Recently, a growing number of therapies have also continuously given special emphasis on virulence factors (Issakhanian and Behzadi, 2019; Sarshar et al., 2020). However, antibiotics remain to be the first-line option for *E. coli–related* UTIs in many countries (Flores-Mireles et al., 2015; Behzadi et al., 2020). Our previous result indicated that multidrug-resistant (MDR) *E. coli* was sensitive to carbapenem but was resistant to most of the antibiotics, which suggests that the choice of antibiotics targeting *E. coli* is extremely limited (Chen et al., 2018). Moreover, overuse and misuse of the antibiotics has resulted in the increasing emergence of multidrug resistance (MDR) *E. coli* strains which have acquired more virulence factors and stronger pathogenicity, thus threatening the utility of carbapenems as reliable agents for UTIs (Jahandeh et al., 2015; Sihra et al., 2018; Vihta et al., 2018; Behzadi, 2020). Therefore, identifying a host-targeted therapy is appealing to provide new options for UTIs and to overcome the resistance of *E. coli* due to the low mutation rate of host genes compared with bacteria.

Traditional Chinese medicine (TCM) has unparalleled advantages of possessing the synergistic action of multiingredient, multitarget, and multichannel medicines, as compared with the single-target drugs, which hence contributes to the further resource of novel therapies against UTIs (Ru et al., 2014; Klein and Hultgren, 2020). In China, Coptis chinensis Franch (CCF), especially the dried root of Coptis chinensis Makino, has been widely used in traditional medicine and food consumption for its functions of clearing heat and detoxification (Meng et al., 2018; Wang et al., 2018). Currently, the compounds in CCF have been reported to exhibit a wide spectrum of pharmacological properties, including antibacterial (Jamshaid et al., 2020; Gaba et al., 2021), anti-inflammatory (Tarabasz and Kukula-Koch, 2020; Gaba et al., 2021), and immunoregulatory effects (Wang et al., 2018; Kwon et al., 2021) among others. Besides, almost most of the articles have constantly given special emphasis on the alkaloids of CCF, especially palmatine, berberine, and berberrubine (Meng et al., 2018; Wang et al., 2018; Jamshaid et al., 2020; Tarabasz and Kukula-Koch, 2020; Gaba et al., 2021; Kwon et al., 2021). Actually, CCF is tackled as a whole herb in traditional Chinese medicine (TCM) for thousands of years. Exploring the potential targets of CCF against UTIs may be more valuable to maintain its overall characteristics. Nevertheless, the traditional research method finds it difficult to decipher the multilevel pharmacological mechanisms of CCF from a systematical perspective. With the constant amendment of the accuracy of biological big data and the deeper intersection of bioinformatics as well as the multipharmacology, the research pattern of “multiingredient, multitarget, and multipathway synergistic actions” of the network pharmacology will be better applied to explore the complicated mechanisms of natural products (Ru et al., 2014; Yang et al., 2019).

**FIGURE 1:**
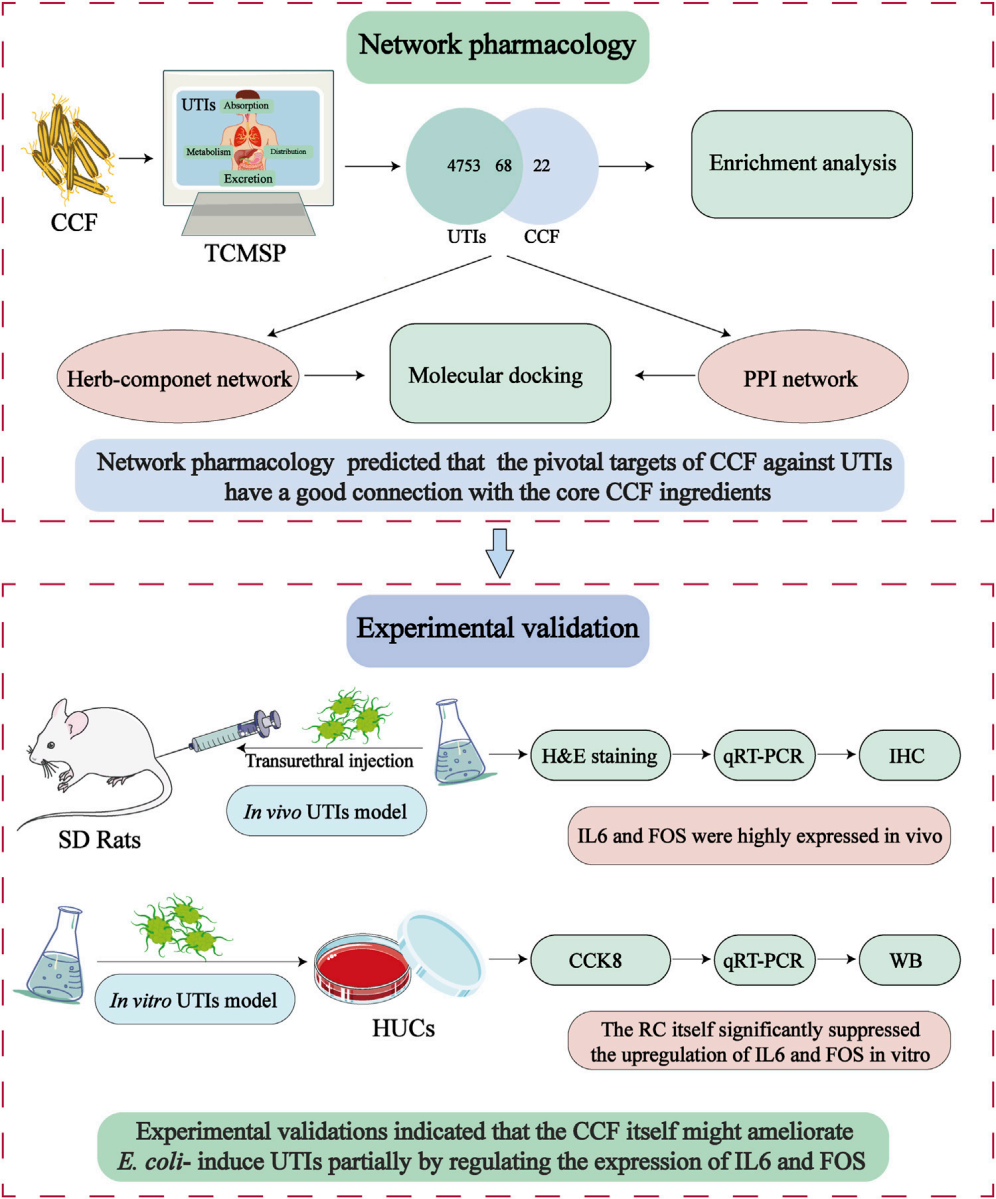
System-pharmacology workflow for the analysis of CCF against UTIs.

In the current research, for the first time, the pharmacological investigation and experimental evaluations on CCF against UTIs were carried out to encode the pivotal targets of action of CCF itself, as shown in Figure 1. Primarily, the pivotal ingredient and potential targets of CCF were predicted by computational tactics. Experimentally, the therapeutic potential and possible targets of CCF itself against UTIs were further verified by *E. coli–induced* UTIs in rats and human uroepithelial cells (HUCs). The obtained data will provide evidence for the therapeutic potential and targets of CCF against UTIs and enrich the medicinal uses of CCF.

## 2 Materials and Methods

### 2.1 Computational Prediction Methods

#### 2.1.1 Screening for the Coptis Chinensis Franch Active Components and Potential Target Genes

The active CCF components and targets were retrieved from the traditional Chinese medicine systems pharmacology (TCMSP) (http://lsp.nwu.edu.cn/tcmsp.php), a database of systems pharmacology for drug discovery from 499 Chinese herbal medicines. The keyword “Coptis chinensis Franch” was utilized for collecting the candidate chemical ingredients. The oral availability (OB) and drug-likeness (DL) were regarded as the core indicators in drug screen, and the constituents with the established criteria of OB ≥ 30% and DL > 0.18 were selected as active components for further study. The chemical structures of the selected active compounds were prepared using ChemDraw.

#### 2.1.2 Prediction of UTI-Related Targets

Using the keyword, urinary tract infection, we searched for UTI-specific union target genes using the therapeutic target database (TTD, http://db.idrblab.net/ttd/), the drug bank database (https://go.drugbank.com/), the online Mendelian inheritance in man database (OMIM, http://omim.org/), the PharmGKB database (https://www.PharmGkb.org/), and the microarray dataset GSE43790 which was downloaded from the GEO database on the basis of the screening criteria of *p* < 0.05. The overlapping disease and drug targets inferred the CCF targets in the treatment of UTIs.

### 2.1.3 The Disease–Target–Component–Herbal Network Construction

Cytoscape 3.7.2 was used to perform network biology analysis and visualization. We then constructed the visual analysis of the drug–ingredient–target–disease interaction network to illustrate the complicated relationships among the disease, genes, drug, and components. The node was used to indicate the candidate compounds and target proteins, while the connection shows the interaction between two nodes.

### 2.2 The PPI Network Construction

To forecast the interactions of the overlapping targets, they were put into the STRING database to construct a PPI network. Here, we limited the species to “Homo sapiens,” and the lowest interaction score was set to a medium confidence (0.700). We eliminated the disconnected targets and then maintained the remaining parameters at default settings. We then used topological analysis plug-in CytoNCA to filter out the key targets based on betweenness centrality (BC) ≥ median BC, closeness centrality (CC) ≥ median CC, degree centrality (DC) ≥ median DC, eigenvector centrality (EC) ≥ median EC, network centrality (NC) ≥ median NC, and local average connectivity–based centrality (LAC) ≥ median LAC (Tang et al., 2015).

### 2.3 Component–Target Molecular Docking

Two-dimensional (2D) structures of the core effective CCF ingredients were downloaded from the PubChem database and saved in the SDF format. The 2D structures were transformed into the mol2 format via Chem3D software and then saved in the PDBQT format as docking ligands using the AutoDock Tool. On the other hand, the core proteins of the overlapping genes with the highest resolution were searched at the Uniport database (https://www.uniprot.org/) and then were regarded as receptors. Besides, the crystal structures of these targets were screened at the RCSB PDB database (https://www.rcsb.org/). We removed the solvent and organic molecules using Pymol software, added nonpolar hydrogen through AutoDock 1.5.6 software, and then saved the structures as PDBQT files (Trott and Olson, 2010). To evaluate the potential affinity, we calculated the docking score by utilizing AutoDock Vina (An et al., 2020). The grid box for IL6 used centers of −0.036, −0.02, and 0.251 with the corresponding sizes of 40, 40, and 40 (x, y, and z positions, respectively) for comprising each amino acid residue of the protein that was simulated (spacing = 0.375). The grid box of FOS used centers of 36.191, 10.912, and −14.602 with the corresponding sizes of 40, 40, and 40 (spacing = 0.375). The grid box of EGFR used centers of 83.758, 56.16, and 50.178 with the corresponding sizes of 40, 40, and 40 (spacing = 0.375). The grid box of MYC used centers of 53.465, 47.64, and 57.415 with the corresponding sizes of 40, 40, and 40 (spacing = 0.375). Although these small molecules predicted by network pharmacology tend to show nonspecific “docking” effects, they could be the primary basis for defining the therapeutic potential of CCF intervention in UTIs.

### 2.4 Enrichment Analysis and Network Construction

To further evaluate the underlying molecular mechanisms of CCF in the treatment of the UTIs, the “Org.Hs.eg.db” and “cluster profiler” packages in R (Version 3.6.2) were used to analyze gene ontology (GO) and Kyoto Encyclopedia of Genes and Genomes (KEGG), and then, the bubble plot was applied to visualize the enrichment results. The GO enrichment analysis analyzed the biological process (BP), cellular component (CC), and molecular function (MF). The threshold for analysis was an adjusted *p*-value < 0.05 and FDR < 0.05. To deeper understand the relationships among CCF, targets, vital pathways, interested biological functions, and UTIs, networks were constructed and visualized with cytoscape software.

### 2.5 Source of Bacterial Strains and Cells

Wild-type urinary tract pathogenic *Escherichia coli* strains and human uroepithelial cell (HUC) lines were purchased from the American Type Culture Collection (ATCC, Manassas, VA). All *E. coli* strains were grown in a terrific broth (TB) medium for 8 h overnight. The required suspension was prepared with PBS for further experiments. HUCs were incubated with WT-CFT073 at a ratio of 1:10.

### 2.6 Preparation of Various Concentrations of Coptis Chinensis Franch

Coptis chinensis Franch (CCF), a commercial extract, was purchased from Yuanye (M06GB147650, Shanghai, China). The various concentrations of CCF were prepared according to the corresponding instructions. The CCF was dissolved in DMSO. After the solutions were ultrasonicated and filtered, the filtered solutions underwent further experiments. The final concentrations of CCF were 0, 3, 10, 30, 100, and 300 mg/L.

### 2.7 UTI’s Animal Model

All animal experiments were consistent with guidelines approved by the Institutional Animal Care and Use Committee at the Second Affiliated Hospital of Guangzhou Medical University (China). A total of 12 female 6-week-old Sprague–Dawley (SD) rats, weighing from 160 to 180 g, were purchased from Guangdong Medical Laboratory Animal Center (Guangdong, China) and lived in a specific pathogen-free environment (SPF) room. The body weights were recorded on the first and third days. The transurethral injection of *E. coli* to induce the UTI model was performed as previously described (An et al., 2021). The 12 rats were randomly divided into two groups as follows: group 1 was the normal control group, while group 2 received *E. coli*. All rats were dissected after 3 days of injection. Meanwhile, bladder tissues were harvested under anesthesia for histological examination and mRNA detection. Subsequently, all urine samples were collected to evaluate the bacteria load by log _10_ colony-forming units (CFUs) per rat. Urine samples of rats were serially diluted and plated onto blood agar plates at 37°C for 12 h.

### 2.8 Quantitative Real-Time PCR

RT-PCR was then applied to evaluate the expression of predicted hub genes in rat bladder tissues that occur after the infection of *E. coli*. The collection of total RNA, primer-specific reverse transcription (RT), and PCR were performed according to the corresponding instructions (TaKaRa Biomedical Technology, Dalian, China). RocheLightCycler480 was used for all data analysis. Based on the 2^−ΔΔCt^ method, the relative expression of IL6, FOS, MYC, and EGFR mRNA was calculated. The detailed primer sequences are shown in Table1.

**TABLE 1:** Sequences of identified four hub genes.

Gene name		Primer sequences (5′-3′)
EGFR	Forward	ATC​TGG​GTA​CGT​TCA​ATG​GCA
	Reverse	TGT​CCA​GTG​GTC​AAC​AAG​GTG
MYC	Forward	AGG​GAG​ATC​CGG​AGC​GAA​TA
	Reverse	GTC​CTT​GCT​CGG​GTG​TTG​TA
IL6	Forward	TGC​AAT​AAC​CAC​CCC​TGA​CC
	Reverse	ATT​TGC​CGA​AGA​GCC​CTC​AG
FOS	Forward	GAC​TGA​TAC​ACT​CCA​AGC​GG
	Reverse	CAT​CAG​GGA​TCT​TGC​AGG​C

### 2.9 Hematoxylin and Eosin and Immunohistochemistry

To observe the histological changes of bladder tissues, all fixed bladder samples were embedded in paraffin and cut into 6 μm sections for hematoxylin and eosin staining. For each HE-stained bladder section, five random fields were scored according to the previous criteria (Liu et al., 2016; He et al., 2020). IL6 and FOS protein expressions in rat bladder tissues were detected by immunohistochemistry. For each IHC-tissue section, five random visual fields were applied for determination. After these tissue sections were dewaxed and rehydrated, the slices were repaired in a boiled sodium citrate buffer (10 mM, pH 6.0) in an electromagnetic oven for 8 min. Subsequently, endogenous peroxidase was removed, and nonspecific binding was blocked for 30 min with serum. Then, the sections were incubated at 4°C overnight with primary antibodies: IL6 (1:200) and FOS (1:300). On the following day, slices were incubated with secondary antibodies and a peroxidase solution, followed by DAB dyeing. When stained brown, the slices were washed with running water to terminate DAB dyeing before hematoxylin staining for approximately 10 s. The subsequent steps were alcohol dehydration, xylene transparency, covering, and observation by using a polarizing microscope (CX31 Olympus, Tokyo, and Japan) and a tissue scanner (PathScope 4s, DigiPath, NV, United States). Finally, we evaluate the relative expression of IL6 and FOS by calculating the means of integrated optical density (IOD) by ImageJ software.

### 2.10 Cell-Counting Kit 8 Assay

A CCK8 Kit (Dojindo Laboratories, Tokyo, Japan) was applied to evaluate the cell viability rates. The cells were seeded in 96-well plates at a density of 4*10^3^ cells/well at 37°C and cultured for 48 h. Then, the HUCs were washed with PBS thrice, followed by incubation with 0.1% DMSO and various concentrations of CCF ranging from 0 mg/L to 300 mg/L (Yuanye, Shanghai, China) for 3 h. Afterward, a CCK8 working solution was added. After incubation for an additional 4 h at 37°C, a hybrid multimode microplate reader (Bio-Tek, Vermont, United States) was used for detecting the OD values at 450 nm.

### 2.11 Western Blot

HUCs were collected after incubation for 3 h with or without *E. coli* in the presence or absence of CCF (3 mg/L). Cell samples were washed by PBS and lysed in RIPA buffer. After the lysates were ultrasonicated and centrifuged, the supernatants were retained. The protein concentrations were evaluated by using a bicinchoninic acid assay (BCA) protein assay kit (Thermo Fisher Scientific, Waltham, MA). All proteins were denatured by heating before equal amounts of protein were added to the chamber for electrophoresis and transferred to an NC membrane (Millipore). After being blocked by 5% skimmed milk for 1–2 h, the membranes were incubated at 4 °C overnight with primary antibodies: IL6 (1:1,000), FOS (1:1,000), and GAPDH (1:1,000). After being washed in TBST three times, the membranes were incubated with the secondary antibodies (Santa Cruz Biotechnology) for 1–2 h at room temperature. The ImageJ software (Rawak Software Inc., Germany) was applied to quantify the relative band density.

### 2.12 Statistical Analysis

All results were analyzed by using SPSS software version 20.0. The value of *p* < 0.05 was considered statistically significant. The CCK8 assay was conducted in quintuplicate and repeated three times. The entire experiment of PCR, WB, H&E-staining inflammatory scores, and IHC were performed in six biological and technical replicates. The statistical significance of bacterial burden in urine, H&E-staining inflammatory scores, and IHC was assessed by t-tests. The statistical significance of CCK8 and WB were assessed by one-way ANOVA with LSD post hoc comparisons or Dunnett’s T3 post hoc tests. The statistical significance of PCR was assessed by t-tests or one-way ANOVA with LSD post hoc comparisons or Dunnett’s T3 post hoc tests.

## 3 Results

### 3.1 Acquisition of the CCF Active Compounds and Targets

**FIGURE 2:**
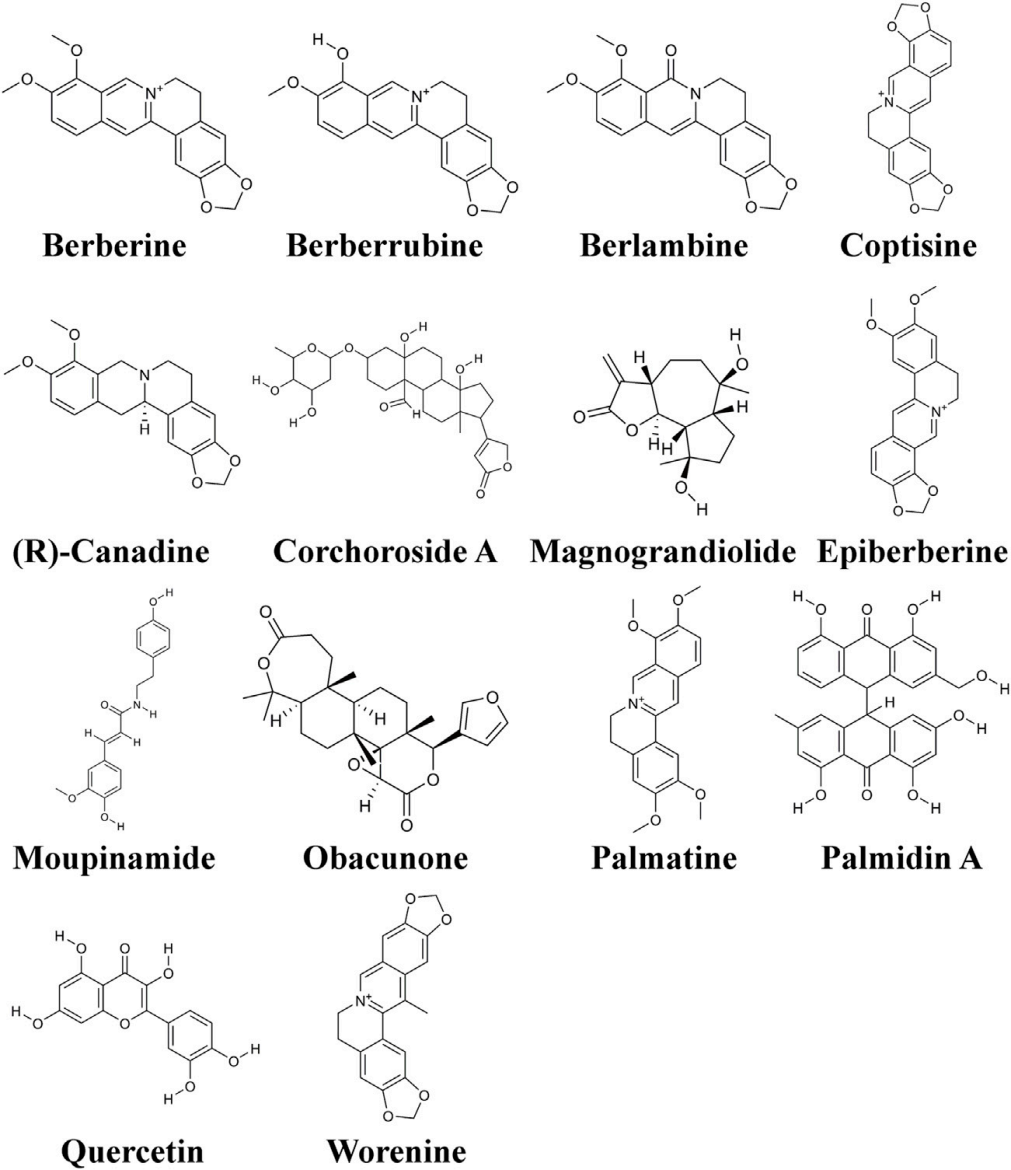
Structures of the 19 active ingredients in CCF.

To sieve key ingredients, we first obtained a total of 48 candidate compounds from the TCMSP database based on the absorption, distribution, metabolism, excretion, and toxicity (ADME/T) values. Using an oral bioavailability (OB) ≥ 30% and a drug-likeness (DL) ≥ 0.18, we selected 14 components as vital effective ingredients (Figure 2). Subsequently, 90 genes which showed potential mediation of these bioactive compounds in the treatment of UTIs were obtained from the TCMSP database.

### 3.2 Prediction of CCF Target Genes in the Treatment of UTIs

**FIGURE 3:**
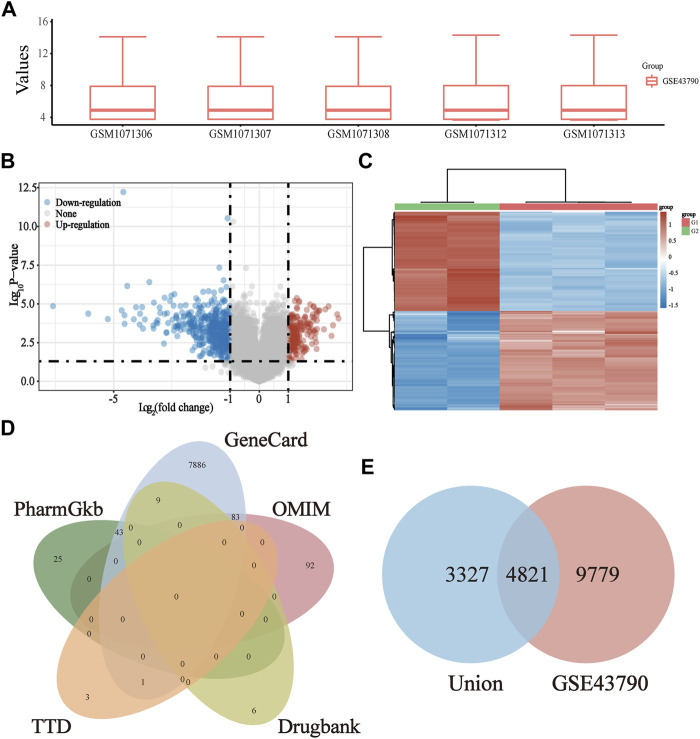
Screening of UTI-specific targets from six databases. **(A)** Box plot after data standardization. Rows refer to samples, and columns indicate gene expression values in the sample; **(B)** Volcano plot for the DEG of UTIs was established using the *p*-value. The red points in the plot mean the upregulated targets, while the blue points represent the downregulated genes with statistical significance; **(C)** hierarchical clustering analysis of targets, which were differentially expressed between model and normal groups; **(D)** screening out the UTI-related union targets using the DrugBank, GeneCards, TTD, OMIM, and PharmGKB databases; **(E)** Venn diagram of the UTI GEO genes and union genes.

The gene expression profile data of the UTIs targets were obtained using GSE43790 from the GEO database, followed by quality control and standardization of the UTI data (Figure 3A). Afterward, differential expression analysis was conducted by using the “limma” package of the R language (*p*-value <0.05), which yielded a total of 14600 UTI-related GEO genes. Meanwhile, a volcano plot (Figure 3B) and heat map (Figure 3C) were drawn. Subsequently, a total of 8148 UTI-related union target genes were identified from the DrugBank, GeneCards, TTD, OMIM, and PharmGKB databases (Figure 3D). After intersecting the UTI GEO and union genes, 4,821 target genes were identified as the UTI-related target genes (Figure 3E).

### 3.3 Analyses of a Drug–Compounds–Genes–Disease Network

**FIGURE 4:**
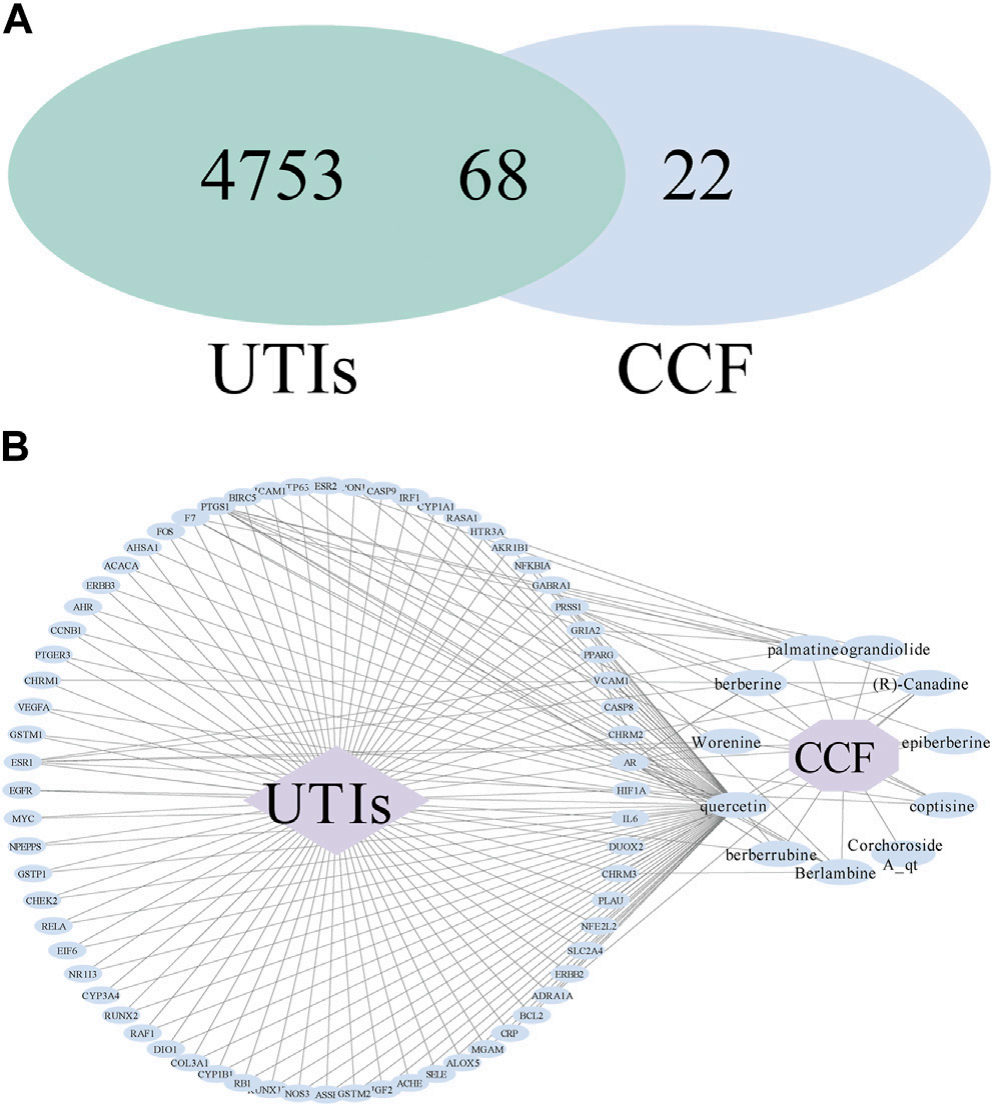
Drug–compounds–genes–disease network. **(A)** Venn diagram of the drug–disease crossover genes; **(B)** drug–compounds–genes–disease network. The blue ellipse nodes indicate the targets or ingredients, the purple diamond indicates the disease, the purple octagon means the drug, and the black line represents the interaction.

After intersecting the disease targets and the obtained drug targets, we constructed a Venn diagram having the intersected targets and 68 common genes (Figure 4A). Based on the target screening data, we constructed a visual analysis of the component-target network using Cytoscape 3.7.2 software (Figure 4B). There were 80 nodes in the network, with 68 overlapping targets, 10 active compounds, one drug, and one disease. The effective ingredients, based on the frequency of target hits, were more likely to have anti-inflammatory properties. Our data showed that quercetin was the principal ingredient, followed by palmatine (R)-canadine, berlambine, berberine, and berberrubine.

### 3.4 The PPI Network Construction and Mining of Core Targets

**FIGURE 5:**
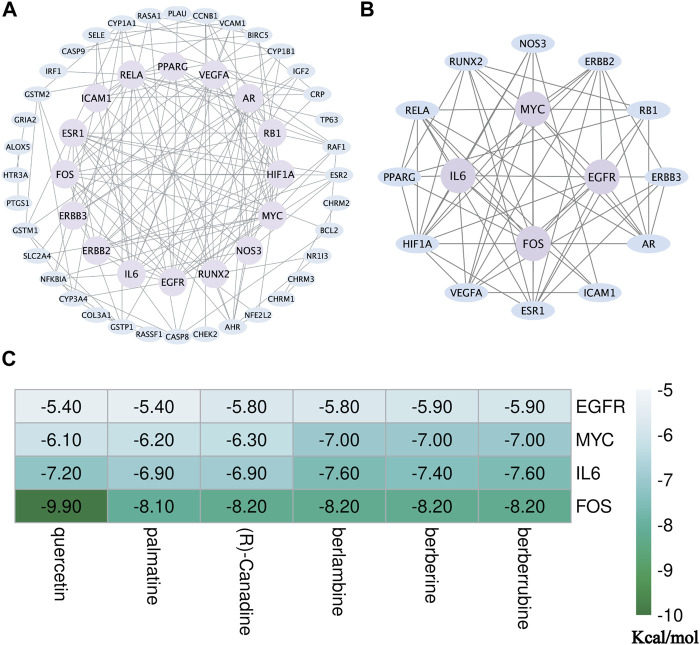
Identification of the core targets of CCF in the treatment of UTIs. **(A)** First PPI network containing 52 nodes and 149 edges was identified via CytoNCA. The blue nodes represent the target genes, while those highlighted in purple represent the selected genes ranked in the top 50% for BC, CC, DC, EC, NC, and LAC; (**B)** second PPI network containing 16 nodes and 62 edges, where the purple areas represent the core targets; **(C)** thermogram shows the docking score of the selected component–target interaction. The greener the color of the term, the greater the possibility of the molecular interaction.

To have a better understanding of the sophisticated functional networks between the overlapping targets, we imported 68 CCF target genes into the STRING database, removed isolated nodes, and then successfully constructed a PPI network (Figure 5A). The network comprised 52 nodes and 149 edges. To screen out key targets, the BC, CC, DC, EC, NC, and LAC of each node were calculated using the CytoNCA plug-in in Cytoscape. Having BC ≥ 11.57548171, CC ≥ 0.1059191175, DC ≥ 5, EC ≥ 0.0749925375, NC ≥ 2.2, and LAC ≥3, we obtained 16 candidate genes which were used for further topological analysis. A total of four core target proteins, namely, IL6, FOS, MYC, and EGFR, were selected by the calculated median of the topological parameter (BC = 76.15148495, CC = 0.111720812, DC = 10.5, EC = 0.231063858, NC = 4.176923077, and LAC = 5.8615079365) (Figure 5B). These data indicated that IL6, FOS, MYC, and EGFR may be the underlying targets of action of CCF against UTIs.

### 3.5 Molecular Docking of the Vital Target Molecules

To better understand the interaction between the six vital targets and the six core active ingredients, we performed docking to explore the best binding position. We obtained the crystal structures of interleukin-6 (1ALU), protein-oncogene c-FOS (2WT7), MYC proto-oncogene (5I50), and the epidermal growth factor receptor (5LV6) from the RCSB PDB database. In the process of preparing proteins, we removed the water molecules and irrelevant ligands, followed by the addition of hydrogen atoms. Subsequently, AutoDock Vina was applied to calculate the docking scores representing the potential affinity. The lower the energy, the more stable the interaction. The potential affinities for the six ingredients (quercetin, palmatine (R)-canadine, berlambine, berberine, and berberrubine) with the IL6 crystal structure were −7.2, −6.9, −6.9, −7.6, −7.4, and −7.6 kcal/mol, respectively. The docking scores for the four ingredients with the FOS crystal structure were −9.9, −8.1, −8.2, −8.2, −8.2, and −8.2 kcal/mol, respectively. The docking scores for the four ingredients with the EGFR crystal structure were −5.4, −5.4, −5.8, −5.8, −5.9, and −5.9 kcal/mol, respectively. The docking scores for the four ingredients with the MYC crystal structure were −6.1, −6.2, −6.3, −7.0, −7.0, and −7.0 kcal/mol, respectively. Our results indicated that the whole four key proteins had potential affinity to the core components (Figure 5C).

### 3.6 GO Functional Enrichment Analysis for the Identified Overlapping Genes

**FIGURE 6:**
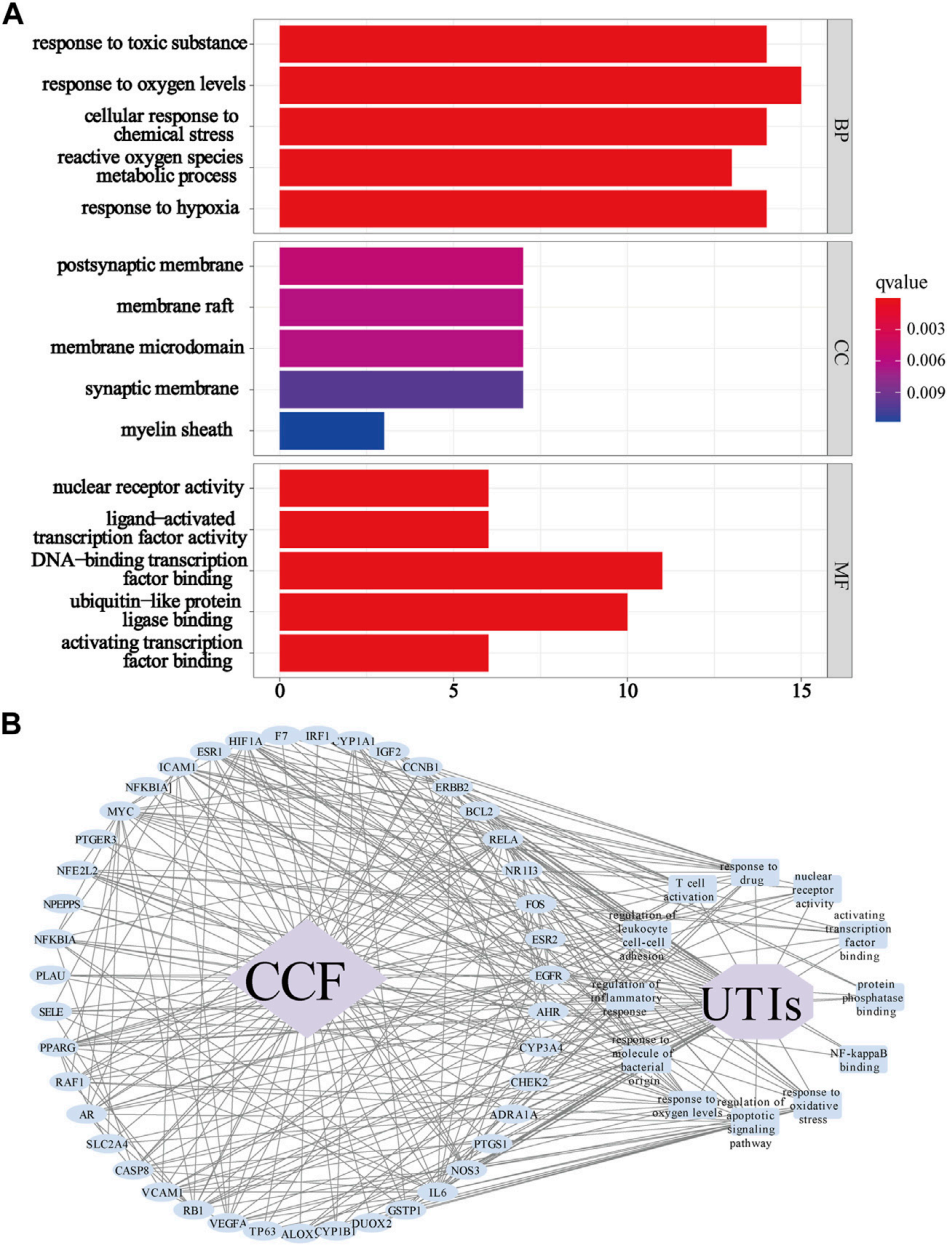
GO enrichment analysis for the identified genes. **(A)** GO analysis showing the biological process, cellular component, and molecular function; Each bar represents a GO term on the *y*-axis, while the horizontal axis indicates the number of proteins enriched in each term. The color shows the adjusted *p*-value of each GO term. The bluer the color of the term, the smaller the p adjusted value. (**B)** Target-biological function network. The blue ellipse nodes refer to the targets, the blue round rectangles show the picked biological functions or signaling pathways, the purple octagon means the disease, the purple diamond indicates the drug, and the black lines represent the interaction.

To identify the differentially affected functions by the 68 overlapping proteins, we performed GO enrichment analysis through R (Version 3.6.2) (Figure 6A). After excluding unrelated terms, 1,133 terms were predicted to be related to BP, mainly involved in responses to oxygen levels (GO:0070482), responses to drugs (GO:0042493), responses to oxidative stress (GO:0006979), regulation of apoptotic signaling pathways (GO:2001233), responses to molecules of bacterial origin (GO:0002237), regulation of inflammatory responses (GO:0050727), regulation of leukocyte cell–cell adhesion (GO:1903037), and T cell activation (GO:0042110). On the other hand, 11 terms were shown to be related to CC, especially in the membrane microdomain, transcription regulator complex, nucleus, and cytoplasm of cells, while 102 terms were related to MF such as nuclear receptor activity (GO:0004879), activating transcription factor binding (GO:0033613), protein phosphatase binding (GO:0019903), and NF-kappa B binding (GO:0051059). The GO analyses indicated that CCF might exert its therapeutic effects on UTIs through participating in numerous biological regulation processes. To shed light on complicated processes of CCF against UTIs, we created a network showing important targets in 12 GO terms of interest mentioned above (Figure 6B). In this network, 57 nodes were interconnected by 315 edges. The above results indicated the characteristics of multilevel biological regulation processes of CCF in the treatment of UTIs.

### 3.7 KEGG Functional Enrichment Analysis and the Protein-Pathway Network

**FIGURE 7:**
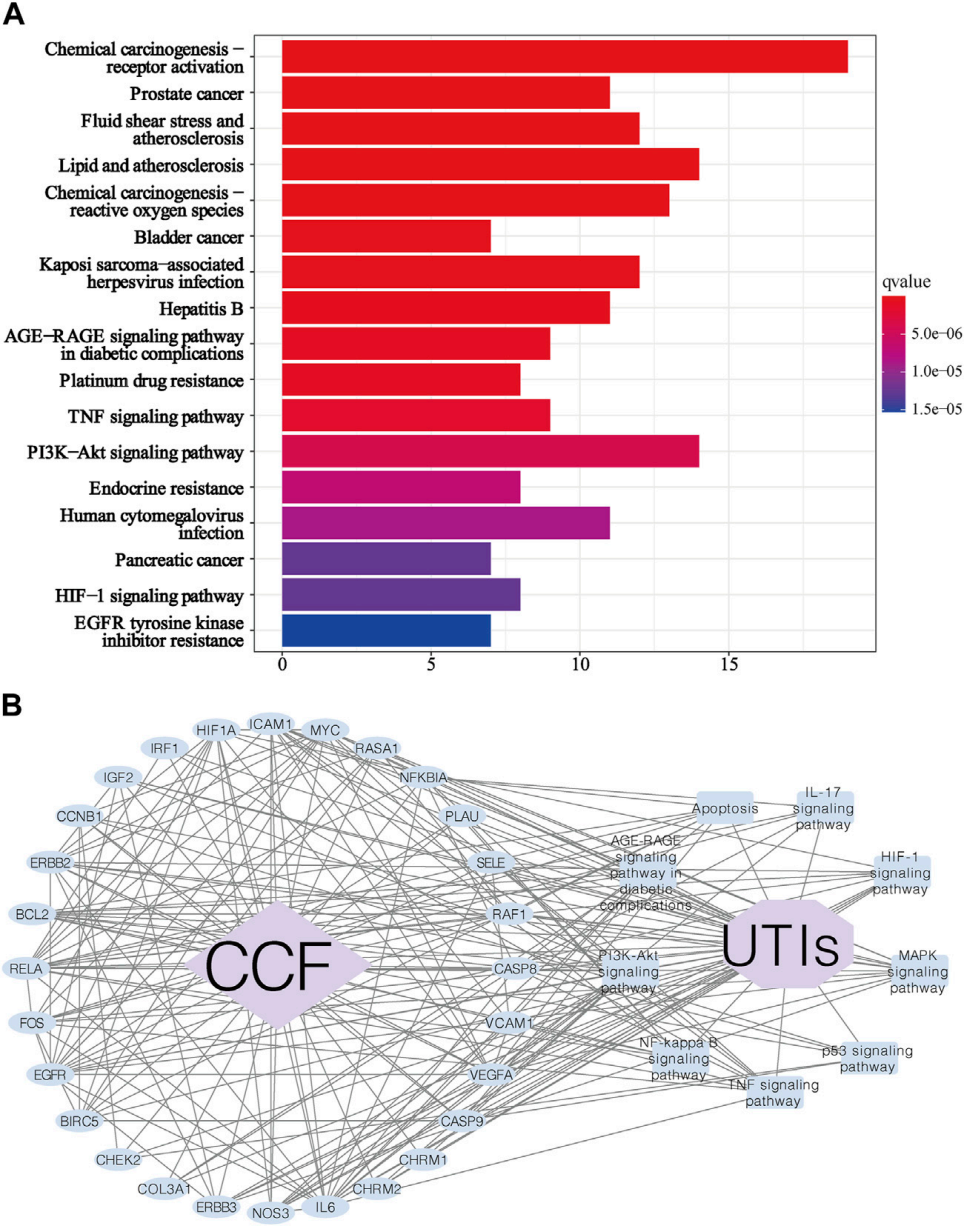
KEGG enrichment analysis for the identified genes. **(A)** Enrichment analysis of the KEGG signal pathway for the potential targets in the treatment of UTIs. Each bar represents a KEGG term on the *y*-axis, while the horizontal axis indicates the number of proteins enriched in each term. The color shows the adjusted *p*-value of each KEGG term. The bluer the color of the term, the smaller the p adjusted value. (**B)** Target-signaling pathway network; the blue ellipse nodes refer to the targets, the blue round rectangles show the picked biological functions or signaling pathways, the purple octagon means the disease, the purple diamond indicates the drug, and the black lines represent the interaction.

In KEGG enrichment analysis, a total of 115 signaling pathways were obtained based on the adjusted *p*-value (value <0.05) and FDR (value <0.05). We excluded unrelated signaling pathways such as “chemical carcinogenesis—receptor activation,” “Proteoglycans in cancer,” and “Fluid shear stress and atherosclerosis”. The overlapping proteins were closely associated with pathways such as the PI3K-AKT signaling pathway (hsa:04151), MAPK signaling pathway (hsa:04010), AGE-RAGE signaling pathway in diabetic complications (hsa:04933), TNF signaling pathway (hsa:04668), HIF-1 signaling pathway (hsa:04066), Apoptosis (hsa:04210), NF-kappa B signaling pathway (hsa:04064), p53 signaling pathway (hsa:04115), and IL-17 signaling pathway (Figure 7A). We can roughly divide these pathways into modules of inflammation, immune responses, and apoptosis. Based on the KEGG analysis, we created a protein-pathway network to further understand their interaction, and the results showed that CCF was directly linked to UTI-related targets, and these proteins were involved in vital UTI pathways, eventually indirectly connecting CCF with UTIs (Figure 7B). Besides, this network had 39 nodes that were interconnected and associated with 209 edges. This network indicated that CCF could exert anti-inflammatory and immunoregulatory effects through multiple targets and multipathway synergistic action.

### 3.8 Construction of a Successful UTI Model by Transurethral Injection of *E. coli*

**FIGURE 8:**
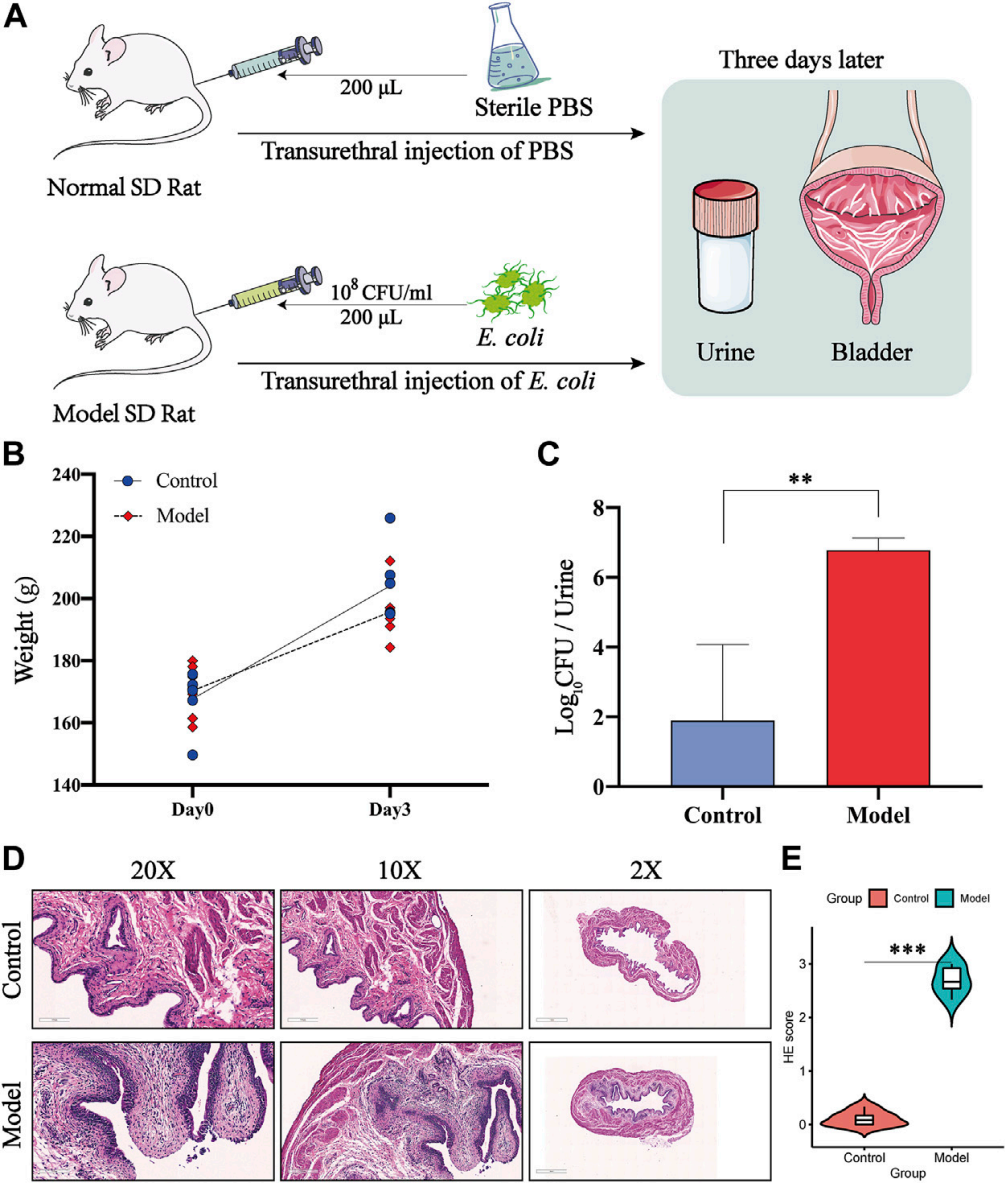
Construction of a successful UTI model by transurethral injection of *E. coli*. **(A)** Animal experimental design of transurethral injection of *E. coli* to induce the UTI model; **(B)** body weights of the control group (*n* = 6) and model group (n = 6); **(C)** level of bacterial burden in urine among two groups; **(D)** representative images of H&E staining of paraffin-embedded bladder sections from two groups; all bladder samples were stained using hematoxylin–eosin (×200 magnification, scale bar:100μm; ×100 magnification, scale bar:200μm; ×2 magnification, scale bar:1 mm); **(E)** semiquantitative data of H&E staining inflammatory scores among groups. All presented values were expressed as means ± S. D, n = 6. **p < 0.01, and ***p < 0.001 compared with the normal control group.

All rats were sacrificed 3 days after transurethral injection of *E. coli* (Figure 8A). To evaluate whether an effective UTI-model was established, we first detected the change of rat weights, and the data showed that there was no significant difference in the body weights among the normal rats and the model rats (Figure 8B). Further, we enumerated the levels of bacterial burden in urine, and the results revealed a high level of bacterial loads caused by the injection of the bacterium compared to the normal group (Figure 8C). To deeper characterize the inflammatory changes of modeling, we further evaluated the histopathological inflammation scores of rat bladders by H&E staining. H&E staining revealed severe inflammatory cell infiltration and loss of the surface epithelium in the infected rats (Figure 8D), and the H&E staining scores of the model group were remarkably increased compared to those of the normal group (Figure 8E). These *in vivo* results indicated that we constructed a successful and suitable model for further investigating the underlying targets of CCF acting on UTIs.

### 3.9 IL6 and FOS Were Highly Expressed in the *E. coli-Induced* UTI Model

**FIGURE 9:**
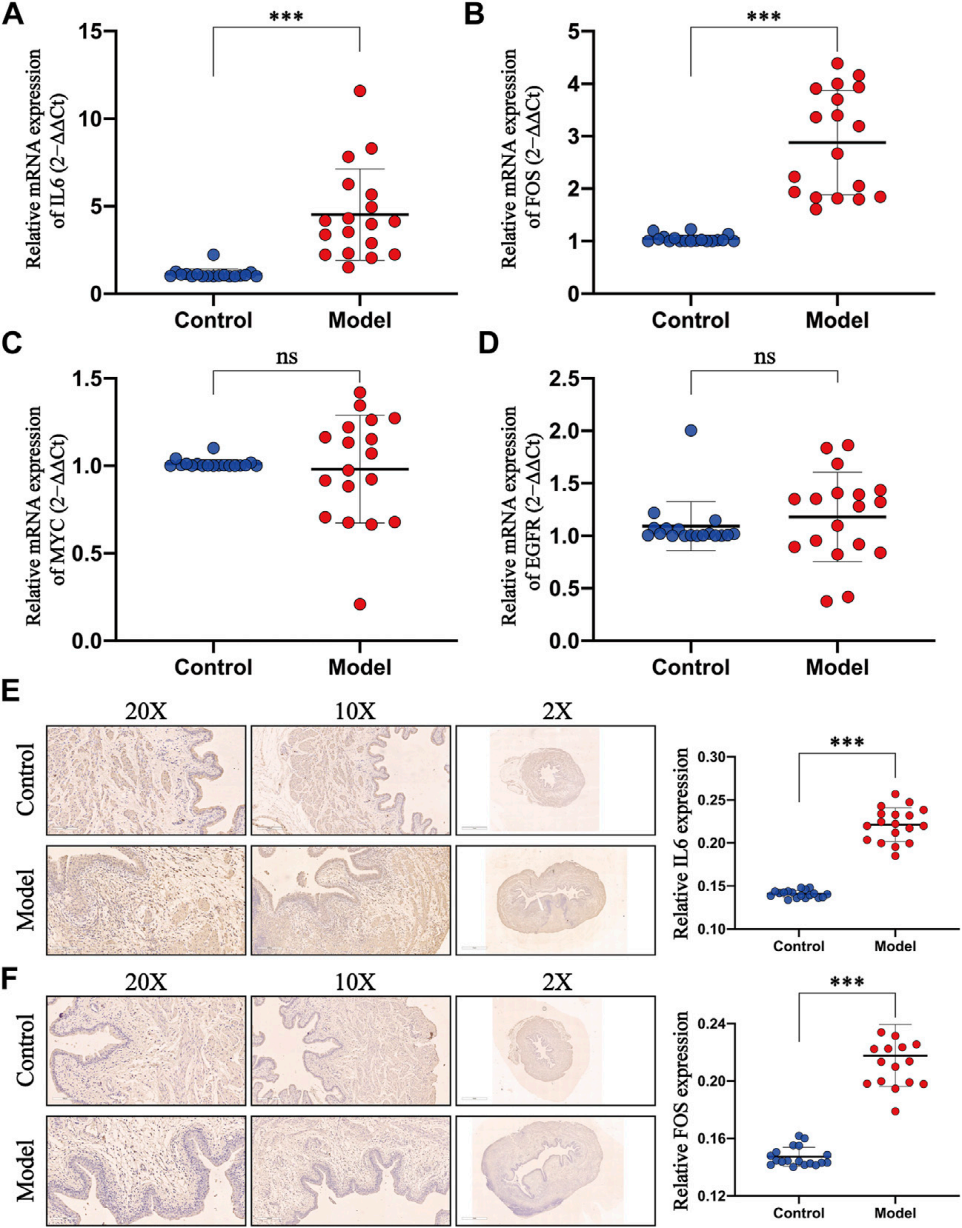
Evaluation of the expression level of pivotal targets in the *E. coli*–induced UTI model. **(A–D)** Levels of IL6, FOS, MYC, and EGFR mRNA were detected by qPCR, and GAPDH was used as an internal control to normalize the data. **(E–F)** Expressions of IL6 and FOS of rat bladders in the control and model groups were evaluated by IHC (×200 magnification, scale bar:100μm; ×100 magnification, scale bar:200μm; ×2 magnification, scale bar:1 mm) and the corresponding semiquantitative data. All presented values were expressed as means ± S. D, *n* = 18. ***p < 0.001 compared with the control group, ns: not statistically significant (Student’s t-test).

Do the pivotal targets, selected by the network pharmacology and bioinformatics analysis, play an important role in the progress of UTIs? This requires experimental evaluations at animal levels. Primarily, we tested the mRNA expression of four core targets in the rat bladders. The detailed primer sequences are shown in Table 1. The results of qPCR indicated that injection of *E. coli* elicited a significant promotion of the mRNA expression of IL-6 (Figure 9A) and FOS (Figure 9B) compared with those of the normal group, whereas the mRNA expression of MYC (Figure 9C) and EGFR (Figure 9D) remained steady. Subsequently, we further evaluated whether the expression of IL-6 and FOS were upregulated using the IHC assay. As predicted, our findings showed that IL-6 and FOS were expressed in agreement with previous observations (Figures 9E,F). These findings indicated that the expressions of IL-6 and FOS were elevated during the occurrence of UTIs.

### 3.10 CCF Intervention Contributes to the Attenuation of *E. coli–Induced* UTIs

**FIGURE 10:**
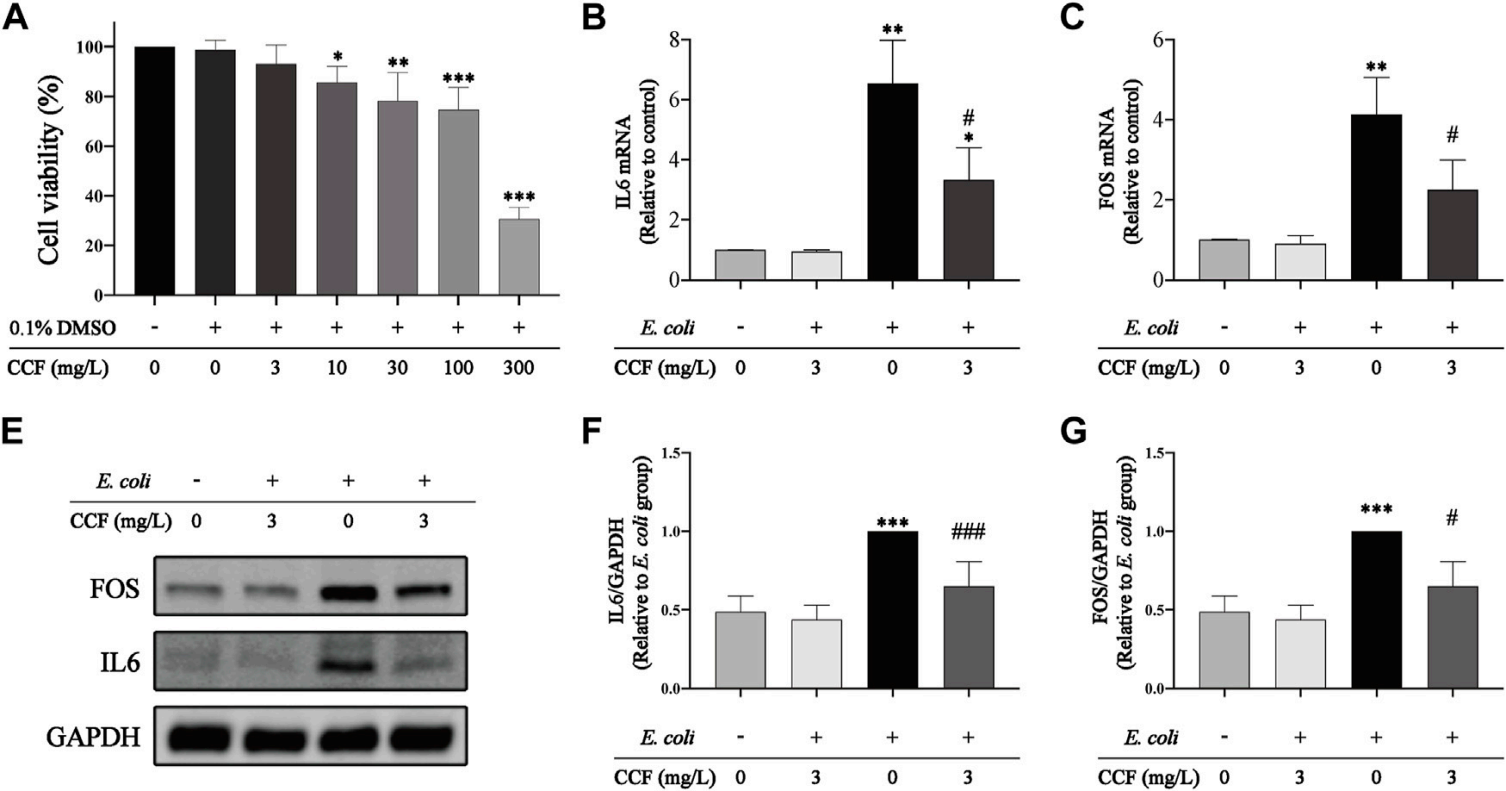
Effect of CCF intervention on cell proliferation and the expression of IL6 and FOS. **(A)** Cell proliferation was examined by the CCK-8 assay; **(B–C)** transcriptional levels of IL6 and FOS were detected by qPCR, and GAPDH was used as an internal control to normalize the data; **(D)** representative WB bands of IL6, FOS, and GAPDH; **(E–F)** statistical analysis of IL6/GAPDH and FOS/GAPDH relative expression. The presented data were presented as means ± S.D., n = 6. *p < 0.05, **p < 0.01, and ***p < 0.001 compared with the control group; #*p* < 0.05, ##*p* < 0.01, and ###*p* < 0.001 versus the *E. coli* alone group, performed by the one-way ANOVA with LSD post hoc comparisons or Dunnett’s T3 post hoc tests.

To further evaluate the anti-inflammatory effect and targets of CCF on *E. coli–induced* UTIs, we performed an *in vitro* experiment using HUCs. We first assessed the cytotoxic effect of CCF on HUCs using the CCK8 assay in various concentrations of it (Figure 10A), and the results revealed a significant reduction in cell viability along with the increase of CCF (≥10 mg/L). Nevertheless, CCF at 3 mg/L showed little effect on the cell viability, which was selected as the suitable concentration for further experiments. Subsequently, we detected the transcriptional level of IL-6 and FOS using the PCR assay. These findings indicated that IL6 (Figure 10B) and FOS (Figure 10C) were highly expressed in HUCs stimulated by *E. coli*, which is further corroborated by our previous *in vivo* results. Moreover, pretreatment with CCF significantly inhibited the upregulation of IL6 and FOS. Last, the protein level of IL-6 and FOS were detected by WB (Figures 10D–F). As expected, a significant reduction of the IL6 and FOS expression could be observed in *E. coli*–stimulated HUCs after the intervention of CCF. Altogether, these results revealed the therapeutic potential and exact targets of CCF acting on *E. coli–induced* UTIs.

## 4 Discussion

In the present study, it was first predicted that IL6, FOS, EGFR, and MYC were the pivotal targets of CCF against UTIs, and they have a good connection with the core CCF ingredients. Afterward, we revealed that IL6 and FOS were obviously upregulated, whereas MYC and EGFR showed no effect in *E. coli*–stimulated rats. Last, the present work demonstrated that CCF alleviates *E. coli–induced* UTIs, at least, partly interceded by restoration of the increased expression of IL6 and FOS *in vitro*.

Urinary tract infections are common recurrent infections with high incidence in both healthy people and patients in hospitals (Vihta et al., 2018). If left untreated or treatment is delayed, the UTIs can lead to permanent damage including kidney parenchymal scarring, renal dysfunction, chronic kidney disease, or death (Foxman, 2003; Hsu and Melzer, 2018). Besides, growing global resistance to antibiotics poses immense challenges for the current UTI treatment strategies (Flores-Mireles et al., 2015; Vihta et al., 2018). Therefore, it is necessary to develop novel host-targeted therapies that would circumvent the resistance to antibiotics. Recent studies have shown that TCM could achieve better therapeutic efficacy as compared to antibiotics (Gharbi et al., 2019; Yang et al., 2019). It is worth noting that network pharmacology analysis is an effective approach in investigating the intricate mechanisms of TCM (Zhao and Iyengar, 2012; Yang et al., 2019). In the current study, a network pharmacology analysis was performed in combination with experimental evaluations to encode the therapeutic potential and targets of CCF against *E. coli* infections.

The disease–drug network analysis of the present study first yielded pivotal and effective CCF components which included quercetin, palmatine (R)-canadine, berlambine, berberine, and berberrubine among others. An interesting study observed that the antiadhesive properties of urine samples were obviously increased after quercetin consumption (Peron et al., 2017). These results of this study are in accordance with the previous published concept of antiadhesive properties of quercetin by forming H-bonds with the FimH protein of *E. coli* (Jaiswal et al., 2018). Palmatine is an anti-inflammation drug in Chinese pharmacopoeia which has been used in augmenting the potency of antibiotics as well (Thakur et al., 2016; Tarabasz and Kukula-Koch, 2020). Further, canadine is a natural alkaloid and is regarded as a novel and promising class of antioxidants and antibacterial drugs (Scazzocchio et al., 2001; Correché et al., 2008). Berberine and its derivatives were also reported to exert various pharmacological activities owing to its antioxidant, immune-modulatory, and anti-inflammatory properties (Ehteshamfar et al., 2020). Moreover, berberrubine, a primary active berberine metabolite, is potentially more pharmacologically active than berberine (Spinozzi et al., 2014). Up to now, the ever-increasing convincing evidence has revealed the numerous pharmacological properties of CCF core ingredients, which suggests that these ingredients may possess any therapeutic relevance. Nevertheless, there are insufficient data on the exact targets of CCF itself against UTIs.

In this study, four hub targets were predicted: IL6, FOS, MYC, and EGFR. IL6 is known as an inflammatory biomarker, while FOS, MYC, and EGFR were originally known as oncogenes. Recently, a growing number of studies have revealed that these oncogenes might play key regulation roles in inflammation and immune responses (Wagner, 2010; Schonthaler et al., 2011; Man and Kallies, 2015; Kim et al., 2018; Fajgenbaum and June 2020). Therefore, the four targets may be responsible for the action of CCF against UTIs. Based on the hypothesis predicted by network pharmacology analysis, the results of RT-PCR and IHC revealed that the mRNA and protein levels of IL6 and FOS were obviously upregulating, whereas MYC and EGFR showed no effect in *E. coli–induced* UTIs, which partially supported the predicted results of network pharmacology analysis. IL6 has been regarded as a pleiotropic proinflammatory cytokine, which is involved in several functions such as maturation of B cells, immunity, inflammation, etc. (Jones and Jenkins, 2018; Kang et al., 2019). Rapid secretions of IL6 are conducive to innate immune response during infection, while the excessive release of IL6 is implicated in the course of inflammatory disorders (Kang et al., 2019). In response to pathogens of UTIs, IL6 is primarily secreted by the host, followed by the release into the serum, thereby inducing a transcriptional inflammatory response through IL6 receptors (Kang et al., 2019; Chamoun et al., 2020; Klein and Hultgren, 2020). The findings of the current study on the elevated levels of IL6 were in consistence with recent studies that also indicated that IL6 is a potent inducer of the progression of UTIs (Becknell et al., 2015; Chamoun et al., 2020; Klein and Hultgren, 2020). Therefore, the expressions of IL6 can be taken as the parameters to judge the effect on the development of UTIs. Furthermore, a growing number of IL6-targeted therapeutic agents have been widely applied for the treatment of numerous acute and chronic inflammatory diseases. Toll-like receptors (TLRs) were regarded as one of the most unique molecules in the progress of UTIs, which can regulate the activation of IL6 (Gluba et al., 2010; Behzadi and Behzadi, 2016; Behzadi et al., 2021a). Unfortunately, TLRs were not included in our study due to the results of the bioinformatics-based network pharmacology. FOS was originally known as an oncogene with functions in controlling the invasive growth and angiogenesis of tumors (Fleischmann et al., 2003). However, the subsequent research studies indicated that FOS can inductively dimerize with members of the JUN family. This hence assembles the transcription factor complex activator protein-1 (AP-1), which plays a key regulation role in inflammation responses (Wagner, 2010; Schonthaler et al., 2011). Similarly, the findings of the present study also demonstrated that FOS is highly expressed in the progression of inflammation. Moreover, innate immune response activated by cytokines could also be regulated by AP-1 (Schonthaler et al., 2011). It is clear that IL6 and FOS could be a potential target of CCF acting on UTIs. The experimental results of RT-PCR and WB analyses in the current study revealed that CCF could inhibit the upregulation of IL-6 and FOS. Therefore, it is evident that CCF contributes to the alleviation of *E. coli-induced* UTIs by regulating the expression of IL6 and FOS.

The *in vitro* bactericidal ability, traditionally, determines the anti-inflammatory capability of drugs. Although it is often difficult to measure the antimicrobial potency of TCM compared with antibiotics, the present work revealed that CCF alleviated inflammation via inhibiting the upregulation of FOS and excessively activated cytokine (e.g., IL6) secreted by the host. Thus, it might provide a new insight to address the current plight of emerging resistance as a result of antibiotic treatment.

## 5 Conclusions

Based on network pharmacology, six core ingredients and four core hub genes of CCF in UTIs were identified in the first phase of the current study. In the next phase, by using *in vivo* experiments, it was found that IL6 and FOS were significantly regulated in UTIs in rats infected by *E. coli*, whereas MYC and EGFR showed no significant difference. Last, it was evident that after treatment with CCF, the disorder of IL6 and FOS can be recovered in *E. coli*–stimulated HUCs. Therefore, the findings of the present study not only revealed the therapeutic targets of CCF but also provided a theoretical basis for CCF against UTIs.

## Data Availability

Publicly available datasets were analyzed in this study. These data can be found at https://www.ncbi.nlm.nih.gov/geo/query/acc.cgi?acc=GSE43790.
